# The association between dexterity and upper limb impairment during stroke recovery

**DOI:** 10.3389/fneur.2024.1429929

**Published:** 2024-08-19

**Authors:** Belen Valladares, Robinson Georg Kundert, Johannes Pohl, Jeremia P. O. Held, Andreas R. Luft, Janne Marieke Veerbeek, Meret Branscheidt

**Affiliations:** ^1^Department of Neurology, University Hospital Zurich, University of Zurich, Zürich, Switzerland; ^2^Cereneo Center for Research and Neurorehabilitation, Vitznau, Switzerland; ^3^Data Analytics and Rehabilitation Technology (DART), Lake Lucerne Institute, Vitznau, Switzerland; ^4^Clinic for Neurology and Neurorehabilitation, Luzerner Kantonsspital, University Teaching and Research Hospital, and University of Lucerne, Lucerne, Switzerland

**Keywords:** rehabilitation, stroke, upper limb, outcome measures, Fugl-Meyer motor assessment, Action Research Arm Test

## Abstract

**Introduction:**

Stroke-induced upper limb disabilities can be characterized by both motor impairments and activity limitations, commonly assessed using Fugl-Meyer Motor Assessment for Upper Extremity (FMMA-UE) and Action Research Arm Test (ARAT), respectively. The relationship between the two assessments during recovery is largely unstudied. Expectedly they diverge over time when recovery of impairment (restitution) plateaus, but compensation-driven improvements still occur. The objective of this study is to evaluate the alignment between FMMA-UE and ARAT in defining upper limb functional recovery categories by ARAT scores. We aimed to establish cut-off scores for both measures from the acute/early subacute, subacute and chronic stages of stroke recovery.

**Methods:**

Secondary analysis of four prospective cohort studies (acute/early subacute: *n* = 133, subacute: *n* = 113, chronic: *n* = 92) stages post-stroke. Receiver operating characteristic curves calculated the area under the curve (AUC) to establish optimal FMMA-UE cut-offs based on predefined ARAT thresholds distinguishing five activity levels from no activity to full activity. Weighted kappa was used to determine agreement between the two assessments. We used minimally clinically important difference (MCID) and minimal detectable change (MDC_95_) for comparison.

**Results:**

FMMA-UE and ARAT scores showed no relevant divergence across all recovery stages. Results indicated similar cut-off scores in all recovery stages with variability below MCID and MDC_95_ levels. Cut-off scores demonstrated robust AUC values from 0.77 to 0.86 at every recovery stage. Only in highly functional patients at the chronic stage, we found a reduced specificity of 0.55. At all other times sensitivity ranged between 0.68 and 0.99 and specificity between 0.71 and 0.99. Weighted kappa at the acute/early subacute, subacute and chronic stages was 0.76, 0.83, and 0.81, respectively.

**Discussion:**

Our research shows a strong alignment between FMMA-UE and ARAT cut-off scores throughout stroke recovery, except among the subgroup of highly recovered patients at the chronic stage. Discrepancies in specificity potentially stem from fine motor deficits affecting dexterity outcomes that are not captured by FMMA-UE. Additionally, the high congruence of both measures suggests they are not suited to distinguish between restitution and compensation. Calling for more comprehensive assessment methods to better understand upper limb functionality in rehabilitation.

## 1 Introduction

Upper limb motor impairments typically reflect deficits in basic body functions, such as the ability to open and close one’s hand, while activity limitations pertain to difficulties in performing daily tasks like drinking from a cup or dressing oneself (1). These two dimensions, although interconnected, do not exhibit a linear relationship between them, suggesting a nuanced relationship between physical impairments and their practical implications during recovery (2–5).

To measure upper limb impairment and activity deficits post-stroke, both the Fugl-Meyer Motor Assessment for Upper Extremity (FMMA-UE) and the Action Research Arm Test (ARAT) have been endorsed by the Stroke Recovery and Rehabilitation Roundtable (SRRR) task force, the European Stroke Organization (ESO) and other international working groups for assessing motor recovery and the quality of rehabilitation (5–8). These high-level recommendations acknowledge FMMA-UE and ARAT as reliable and valid tools for assessing upper limb disability (8–10). The FMMA-UE is designed to capture typical stroke-induced motor impairments, focusing on aspects such as reflexes, coordination, and the ability to move in and out of synergy patterns or produce fractionated movements (11). In contrast, ARAT intends to assess activity limitations in arm usage related to grasping, gripping, and object manipulation (6, 12). Consistency in item response, stability over time and the presence of floor and ceiling effects have been investigated extensively (13–16). However, practical considerations often lead clinical trials to choose either FMMA-UE or ARAT (5). Interestingly, this preference can vary significantly by region, affecting the translatability of trial results (17–20). Additionally, constraints such as patient availability, the need for trained personnel, and cost-effectiveness currently limit the ability of clinical practices to implement both measures outside of research settings.

Therefore, understanding how the results of one domain’s outcome measure might correspond to the context of the other domain remains an intriguing question. Recently, Hoonhorst et al. attempted to delineate this relationship by establishing cut-off scores for FMMA-UE that correspond to clinical ARAT classifications of activity levels (21). They found a high sensitivity and specificity as well as overall agreement for both assessments in five capacity categories. However, their findings were confined to cross-sectional analyses of patients in chronic stages, thereby leaving a substantial gap in our comprehension of this relationship across the entire spectrum of post-stroke recovery. Such understanding is crucial, given the distinct patterns of recovery observed in impairment and activity, as well as the unique aspects of upper limb motor function captured by each assessment tool.

Recovery of impairment, known as true restitution, and recovery of activity exhibit distinct trajectories throughout the post-stroke rehabilitation process (22). Restitution, primarily observed in the initial weeks and months following a stroke, tends to plateau during the subacute stage (23). In contrast, improvements in activity levels can be mediated by compensatory strategies, enabling changes even in the chronic stages after stroke. This divergence suggests that while the restoration of motor function may stabilize relatively early, functional gains in daily activities can continue over a longer period.

Moreover, the assessments of upper limb impairment provided by FMMA-UE and ARAT may not capture all aspects equally. For instance, FMMA-UE subtasks offer only a partial representation of fine motor control, whereas such control significantly influences ARAT outcomes (24). Similarly, while the presence of synergistic movements is not directly evaluated in ARAT scores (12, 25, 26), these movements are assessed with the FMMA-UE. This underscores the importance of considering longitudinal changes in both measures, for better translatability between domains.

Our aim was to evaluate the alignment between FMMA-UE and ARAT in defining upper limb functional recovery categories defined by ARAT scores. We aimed to establish cut-off scores to achieve this alignment by establishing cut-off scores for both outcome measures from the acute/early subacute to the chronic stages of stroke recovery. Specifically, we assessed the agreement of these FMMA-UE thresholds with ARAT’s activity categories—none, poor, limited, notable, and full. We hypothesized a divergence in assessment scores over time, with an initial close alignment between FMMA-UE and ARAT, with both being driven mainly by restitution, and a progressively widening gap as recovery transitions beyond the subacute stage mainly driven by compensation, thus primarily influencing ARAT scores.

## 2 Materials and methods

### 2.1 Study design

We conducted a secondary analysis of existing data pooled from four prospective cohort studies, including one dataset from a rehabilitation inpatient clinic. We selected data at three different stages of recovery: acute/early subacute (2 weeks ± 14), subacute (3–6 months ± 14), and chronic (>6 months) stage post-stroke (27).

Ethical approval from the cantonal ethics committee Zurich and Northwest and Central Switzerland was obtained before study start and adhered to the ethical standards of the revised Declaration of Helsinki. The studies were registered prospectively with their respective BASEC identifiers in Cohort 2 (2017–00948), Cohort 3 (2017–01070), and Cohort 4 (2017–00889). These studies were also registered on ClinicalTrials.gov with the identifiers NCT03294187, NCT03522519, and NCT03287739. All participants or their next of kin provided written informed consent. Secondary data analysis for the cohort 2, 3 and 4 was approved by the cantonal ethics committee Zurich (Business Administrator System for Ethics Committee identifier 2020–00218). Participants from Cohort 1, drawn from a Swiss clinical dataset, cereneo Schweiz AG, consented to further data analysis under the clinic’s general consent agreement (see Supplementary Figure S1). Reporting is adherent to the STROBE reporting guidelines (see Supplementary Table S1) (28).

### 2.2 Study population

The sample comprised stroke patients with a first-ever unilateral ischemic or hemorrhagic stroke, who were recruited at the acute/early subacute, subacute, or chronic stage, were 18 years or older, and had an upper limb motor deficit at the acute stage. Inclusion was based on either FMMA-UE, ARAT or National Institutes of Health Stroke Scale (NIHSS) arm score ≥ 1 motor scores. Participants were excluded if they had a neurological or other disease affecting the upper limb before the stroke, known or suspected non-compliance, drug or alcohol abuse. Patients underwent medical and rehabilitation therapies following Swiss national standards (29). More detailed information regarding specific inclusion and exclusion criteria is provided in the Supplementary material (30–32).

### 2.3 Outcome measures

The FMMA-UE and the ARAT are both recommended outcomes for evaluating upper limb deficits in the International Classification of Functioning, Disability and Health (ICF) body function and activities domain respectively, following International Stroke Recovery and Rehabilitation Alliance (ISSRA) recommendations (1, 5, 7, 8).

#### 2.3.1 Fugl-Meyer motor assessment for upper extremity

The FMMA-UE measures capacity-based impairment in stroke patients. It evaluates movement, coordination, and reflexes in the upper limb using 33 items based on Twitchell and Brunnstrom’s motor recovery phases (33, 34). Higher scores on the 3-point scale indicate more robust motor function, where 0 = cannot perform; 1 = performs partially; and 2 = performs fully, with a maximal total score of 66 points (see Supplementary material) (11).

#### 2.3.2 Action Research Arm Test

The ARAT is an observational measure with 19 items that assess upper limb activity by evaluating the ability to handle objects of different sizes, shapes, and weights to complete activities of daily living tasks. Each item is on a 4-point ordinal scale (0 = unable; 1 = partial; 2 = abnormal; 3 = normal), divided into 4 domains (i.e., grasp., grip, pinch, and gross movement). The maximum score is 57 points, indicating full upper limb activity and the minimum is zero, indicating no upper limb activity (see Supplementary material). The ARAT equipment kit used was the same as in the original article proposed by Lyle (35), and we followed the standardized methodology for conduction and scoring by Yozbatiran (12).

Building upon previous research (21, 36–38), upper limb activity was classified into five distinct categories to allow for comparison with the predefined ICF levels of regained motor function. These categories range from “no activity” (ARAT 0 to 10 points, indicating complete activity limitation according to the ICF) to “full activity” (ARAT 55 to 57 points, indicating no activity limitation and near full recovery). The other categories include “poor activity” (ARAT 11 to 21 points, severe activity limitation), “limited activity” (ARAT 22 to 42 points, moderate activity limitation), and “notable activity” (ARAT 43 to 54 points, mild activity limitation).

### 2.4 Data collection

ARAT and FMMA-UE were administered by trained practitioner-researchers using a standardized protocol (12, 39). Study visits occurred during in-patient hospitalization. After discharge, the patient was evaluated during an out-patient visit or at home (30–32).

### 2.5 Data analysis

Clinical and demographic characteristics were analyzed using descriptive and nonparametric inference statistics (median, interquartile and frequencies) suited to our data distribution. The Kruskal-Wallis test was utilized to analyze ordinal and continuous variables across multiple independent groups, making it ideal for assessing quantitative data that do not follow a normal distribution. Additionally, the Pearson chi-square test was used to analyze differences in nominal variables, which is ideal for testing the independence of categorical data.

To identify optimal FMMA-UE cut-off scores for the five distinct activity categories of the ARAT, we computed receiver operating characteristic (ROC) curves at each stage of stroke recovery for each group separately (acute/early subacute, subacute, chronic) (21, 37). The optimum area under the curve (AUC) in terms of sensitivity and 1-specificity was then identified, and ROC coordinate points were used to select the best threshold based on the maximum sensitivity and 1-specificity (40). For sensitivity and specificity, a test performing below 0.7 is considered unreliable, while a range of 0.7 to 0.9 is considered good, and above 0.9 is excellent (41). To evaluate a test’s accuracy in identifying both false positives and false negatives, the sum of sensitivity and specificity serves as a useful guideline. High-quality testing requires that this combined score is at least 1.5, indicating higher accuracy. A score of 1 renders the test meaningless, while a score of 2 represents a perfect test. Weighted kappa was determined to measure the agreement between the FMMA-UE observations within the five ARAT subcategories (42). Kappa values were interpreted as follows: poor (*k* = 0–0.40), fair (*k* = 0.41–0.75), or excellent (*k* = 0.76–1) (43). We analyzed the differences of FMMA-UE cut-off scores across acute/early subacute, subacute, and chronic stroke recovery groups between our and previously reported results, using established clinically meaningful differences as a reference. These were defined as 13 points for acute/early subacute (44), 9 points for subacute (45), and 5.3 points for chronic stages (46), based on established Minimally Clinically Important Difference (MCID) values. Additionally, we used established values for Minimal Detectable Change (MDC) at a 95% confidence level (MDC_95_) as the minimal difference which could be explained by random variability in the measurements (47). The different cut-off scores across functional recovery categories and chronic stages were compared against these change indicators; only differences exceeding these thresholds were considered relevant.

RStudio software with R version 4.0.3 and IBM® SPSS® Statistics 29 were used for the statistical analyses, and the level of statistical significance was set to <0.05 (48).

## 3 Results

We analyzed data concerning upper limb deficits in patients evaluated with the FMMA-UE and ARAT across the three distinct stages of post-stroke recovery. The acute/early subacute stage included patients assessed within two weeks, plus or minus fourteen days, post-stroke (*n* = 133), where the median assessment time was 9 days (range: 6–30 days). The subacute stage encompassed assessments from 2 weeks before 3 months up to 6 months post-stroke (*n* = 113), with a median assessment time of 93 days (range: 76–163 days). The chronic stage involved patients assessed beyond 6 months post-stroke (*n* = 92), with a median assessment time of 375 days (range: 182–4,850 days). See Table 1 for further patient characteristics. No significant difference was observed in sex, age, limb affected side and distribution of upper limb activity categories, as revealed by Pearson chi-square test or Kruskal-Wallis test. However, a significant difference in ARAT and FMMA-UE total scores, as revealed by the Kruskal-Wallis test, was found between recovery stages, with chronic patients having higher scores than acute/early subacute patients.

**Table 1:** Patients’ characteristics.

Patients’ characteristics	Acute/Early subacute (*N* = 133) C1, *n* = 30 C2, *n* = 0 C3, *n* = 88 C4, *n* = 15	Subacute (*N* = 113) C1, *n* = 9 C2, *n* = 2 C3, *n* = 84 C4, *n* = 18	Chronic (*N* = 92) C1, *n* = 7 C2, *n* = 42 C3, *n* = 43 C4, *n* = 0	*p-*value
Sex, male/female^*^	80/53	64/49	54/38	0.86
Age, years^†^	68.8 ± 14.9	71.4 ± 13	68 ± 12.3	0.07
Limb affected side Left, right	80/53	66/47	55/37	0.96
ARAT total score^‡^	32(0–44)	38(17–55)	39(15–55)	0.001
FMMA-UE total score^‡^	37(13–50)	44(27–56)	41(24–54)	0.006
Upper limb activity categories	Patient *N* = 133	Patient *N* = 113	Patient *N* = 92	0.13
No (ARAT score 0–10)	46	24	20	
Poor (ARAT score 11–21)	9	6	7	
Limited (ARAT score 22–42)	40	33	30	
Notable (ARAT score 43–54)	19	21	11	
Full (ARAT score 55–57)	19	29	24	

Baseline characteristics of all the patients included in the study. Values are ^*^n, ^†^mean ± SD, or ^‡^median (interquartile range). ARAT (Action Research Arm Test). FMMA-UE (Fugl-Meyer motor assessment for upper extremity). C1 = Cohort 1, C2 = Cohort 2, C3 = Cohort 3, C4 = Cohort 4.

Optimal FMMA-UE cut-off scores based on five distinct ARAT activity categories across acute/early subacute, subacute, and chronic stages post-stroke are shown in Table 2. The first row shows ARAT scores for its five recovery categories. Second to Fourth rows show the computed optimal cut-offs for FMMA-UE.

**Table 2:** Optimal FMMA-UE cut-off scores based on five distinct ARAT activity categories across acute/early subacute, subacute, and chronic stages post-stroke.

Upper limb						
Assessment
Activity category	No	Poor	Limited	Notable	Full
ARAT		0–10	11–21	22–42	43–54	55–57
**FMMA-UE**						
Acute/early subacute stage		0–19	20–32	33–47	48–57	58–66
Subacute stage		0–19	20–30	31–47	48–55	56–66
Chronic stage		0–19	20–28	29–45	46–53	54–66
Chronic stage (Hoonhorst et al)^*^		0–22	23–31	32–47	48–52	53–66

^*^Results from Hoonhorst and colleagues for comparison (21).

We compared the results from Hoonhorst et al. on chronic patients to our results. Both results are listed side by side in Table 2 (21). The differences between the FMMA-UE scores defining different activity categories in their study compared to ours ranged from 0 to 3 points across all activity categories at the chronic stage. These differences are below the MCID threshold of 13 points, indicating that they were not clinically relevant. Furthermore, these differences were smaller than the MDC in upper extremity FMMA-UE (MDC_95_) of 5.3 points (44, 46, 47).

We observed only small differences in the FMMA-UE cut-off scores within each upper limb activity category (Table 2). These differences were below the MCID and MDC_95_ threshold, indicating there were clinically insignificant. No Activity: Scores consistently ranged from 0–19 across all stages. Poor Activity: Scores slightly decreased from 20–32 at the acute/early subacute stage to 20–30 at the subacute stage and further to 20–28 at the chronic stage. Limited Activity: Scores varied from 33–47 at the acute/early subacute stage, to 31–47 at the subacute, slightly reduced to 29–45 at the chronic stage. Notable Activity: Scores decreased from 48–57 at the acute/early subacute stage to 48–55 at the subacute and narrowed further to 46–53 at the chronic stage. Full Activity: Scores changed from 58–66 at the acute/early subacute stage to 56–66 at the subacute and adjusted down to 54–66 at the chronic stage.

Table 3 presents the optimal FMMA-UE cut-off scores based on five distinct ARAT activity categories at the acute/early subacute, subacute, and chronic stage post-stroke alongside their corresponding Sensitivity, Specificity and AUC values. At the acute/early subacute stage, AUC ranged from 0.77 (95% confidence interval CI, 0.61–0.93; *p* < 0.004) to 0.92 (95% CI, 0.84–1; *p* < 0.000). At the subacute stage AUC ranged from 0.86 (95% CI, 0.75–0.97; *p* < 0.0001) to 0.94 (95% CI, 0.89–1; *p* < 0.0001). At the chronic stage, AUCs ranged from 0.74 (95% CI.57–1; *p* < 0.024) to 0.93 (CI, 0.83–1; *p* < 0.001). The data in Table 3 demonstrates that the optimal FMMA-UE cut-off scores provide robust diagnostic performance across all stages of post-stroke recovery, evidenced by high sensitivity and specificity values, indicating high accuracy with a low rate of false negatives and positives. An exception to this robust diagnostic performance can be seen at the chronic stage between the categories ‘notable’ versus ‘full’, where specificity was 0.55 and the sum of sensitivity and specificity was 1.38.

**Table 3:** Sensitivity, specificity and area under curve for the computed cut-off scores.

	Activity category	ARAT cut-off	FMMA-UE cut-off	Sens	Spec	Sens + Spec	AUC	AUC 95% CI	*p*-value
Acute/early subacute stage	No *vs* Poor	10	19	0.78	0.87	1.65	0.87	0.77–0.99	<0.0001
	Poor *vs* limited	21	32	0.70	0.99	1.69	0.92	0.84–1	<0.0001
	Limited *vs* Notable	43	47	0.79	0.80	1.59	0.87	0.79–0.96	<0.0001
	Notable *vs* Full	55	58	0.68	0.84	1.52	0.77	0.61–0.93	0.004
Subacute stage	No *vs* Poor	10	19	0.99	0.83	1.82	0.91	0.81–1	0.002
	Poor *vs* limited	21	30	0.82	0.99	1.81	0.91	0.82–1	0.002
	Limited *vs* Notable	43	47	0.86	0.91	1.77	0.94	0.89–1	<0.0001
	Notable *vs* Full	55	56	0.83	0.81	1.64	0.86	0.75–0.97	<0.0001
Chronic stage	No *vs* Poor	10	19	0.99	0.85	1.84	0.93	0.83–1	0.001
	Poor *vs* limited	21	28	0.90	0.71	1.61	0.86	0.71–1	0.003
	Limited *vs* Notable	43	45	0.91	0.80	1.71	0.91	0.80–1	<0.0001
	Notable *vs* Full	55	54	0.83	0.55	1.38	0.74	0.57–1	0.024

The ARAT is scored from 0 to 57 points, and the FMMA-UE is scored from 0 to 66. No activity, ARAT 0 to 10 points; Poor activity, ARAT 11 to 21 points; Limited activity, ARAT 22 to 42 points; Notable activity, ARAT 43 to 54 points; Full activity, ARAT ≥ 55 points. Sens., sensitivity; Spec., specificity; AUC, Area under the curve; CI, confidence interval.

Each cell in Table 4 displays the percentage of patients classified from no activity to full activity by both assessments. Green highlighting indicates a congruent classification between the ARAT activity category and the FMMA-UE activity category. A one-category disagreement, such as from No to Poor or from Notable to Full, results in a yellow highlight. A two-category disagreement is highlighted in red.

**Table 4:** ARAT and FMMA-UE categories agreement at acute/early subacute, subacute and chronic stage post-stroke.

		Activity category	FMMA-UE					
			No (%)	Poor (%)	Limited (%)	Notable (%)	Full (%)	Total (%)
ARAT	Acute/Early subacute	No (%)	27.1	6	1.5	0	0	34.6
		Poor (%)	1.5	5.3	0	0	0	6.8
		Limited (%)	0.8	8.3	15	4.5	1.5	30.1
		Notable (%)	0	0	3	8.3	3	14.3
		Full (%)	0	0	0.8	3.8	9.8	14.3
		Total (%)	29.3	19.5	20.3	16.5	14.3	100
	Subacute	No (%)	17.7	2.7	0.9	0	0	21.2
		Poor (%)	0	5.3	0	0	0	5.3
		Limited (%)	0	8	18.6	2.7	0	29.2
		Notable (%)	0	0	2.7	9.7	6.2	18.6
		Full (%)	0	0	0.9	1.8	23	25.7
		Total (%)	17.7	15.9	23	14.2	29.2	100
	Chronic	No (%)	18.5	3.3	0	0	0	21.7
		Poor (%)	0	5.4	2.2	0	0	7.6
		Limited (%)	0	4.3	22.8	4.3	5.4	32.6
		Notable (%)	0	0	2.2	4.3	5.4	12
		Full (%)	0	0	2.2	2.2	21.7	26.1
		Total (%)	18.5	13	29.3	10.9	28.3	100

Heatmap of activity categories agreement between the ARAT and FMMA-UE scores. Each cell displays the percentage of patients classified from no activity to full activity by both assessments. Green highlighting indicates the matrix diagonal, where both scores agree. Disagreements are highlighted in yellow (discrepancy in one category) or red (discrepancy in two categories).

Table 4 displays a heatmap of activity categories agreement between the ARAT and FMMA-UE scores, at the acute/early subacute stage, perfect agreement (matrix diagonal highlighted in green) accounted for 65.5% of patients, while 4.6% presented major disagreement. At the subacute stage, perfect agreement accounted for 74.3% of patients, and major disagreement occurred in 1.8%. At the chronic stage, perfect agreement accounted for 72.7%, while major disagreement occurred in 7.6%.

The agreement between the five ARAT upper limb activity categories and the associated FMMA-UE scores demonstrated excellent consistency, as evidenced by high weighted kappa results at all stages: Acute/early subacute: weighted *k* = 0.76; 95% CI, 0.69–0.82, Subacute: weighted *k* = 0.83; 95% CI, 0.77–0.89 and Chronic: weighted *k* = 0.81; 95% CI, 0.74–0.88.

## 4 Discussion

Our study aimed to systematically evaluate the alignment between FMMA-UE and ARAT in defining upper limb functional recovery categories across different stages of stroke recovery. We anticipated that the congruence between FMMA-UE and ARAT cut-off scores would vary throughout the recovery process. To explore this hypothesis, we built upon the work of Hoonhorst et al. (21), who investigated a similar relationship but focused on the chronic stage of stroke recovery. In contrast, our study sought to extend this analysis across acute/early subacute, subacute, and chronic stages.

Our findings revealed highly corresponding cut-off scores for the FMMA-UE in relation to upper limb activity categories across all stages of stroke recovery. The differences observed remained within a narrow range, not exceeding established MCID values, which range from 5.3 for chronic to 13 for acute/early subacute stroke patients (44, 46), and an MDC_95_ of 5.2 (47). The overall fit of cut-off scores, reflected in high sensitivity, specificity, and AUC values across all stroke stages, indicates that motor scores achieved on the FMMA-UE are highly associated with the measured activity categories following the ARAT throughout recovery.

As mentioned above, both the ARAT and FMMA-UE are widely used assessments of upper limb function after stroke, measuring different aspects of disability. Current research underscores the difficulty of comparing studies using different scoring systems (4), and recent recommendations (8) suggest using both scores in post-stroke recovery assessments. However, many studies and clinical sites still employ only one measure, likely due to logistical constraints and to minimize effort for both patients and personnel (49–51). This makes it challenging to compare results across studies. Our analysis of categorical agreement between FMMA-UE and ARAT scores at the acute/early subacute, subacute, and chronic stages provide the ability to compare data across studies and clinical sites where only one measure may be available.

Interestingly and similar to Hoonhorst et al., we found a decreased specificity between the FMMA-UE cut-off scores and ARAT categories at the higher ranges, specifically in the “notable versus full” categories at the chronic stage. This divergence between scores at high functional levels suggests that, even with a perfect FMMA-UE score, a remaining deficit may prevent reaching full activity potential. This may be best explained by the fact that fine motor skills are underrepresented in the FMMA-UE assessment, despite being important for achieving full ARAT scores (24).

These results do not imply the redundancy of either assessment tool. The FMMA-UE and the ARAT have previously shown to be highly correlated; however, they are designed to measure different concepts: body impairment versus activity limitation. This is supported by studies that have shown dissociations between these measures, with improvements in one measure but not the other [e.g., (52–54)]. As summarized by Demers and colleagues, movement can be classified at two levels: end point movement in external space (measured by variables such as trajectory speed, precision, and straightness) and movement in body space (measured by variables such as joint ranges, interjoint coordination, and muscle activation patterns) (55). Improvements in end point characteristics can occur through either compensation (e.g., trunk movement to assist reaching) or true recovery of movement in body space. Only movement quality variables in body space can distinguish whether recovery or compensation has occurred.

The ARAT includes speed as a core component, an end point characteristic that can be improved by either restoration or compensation. The test deducts points for compensatory movements, meaning an item cannot receive the full score (three points) if compensatory movements are involved. Furthermore, a score of two is given in the presence of slower movements and/or in the presence of compensatory movements. As such, it does not differentiate well between these factors. Lower scores of one or zero points are given if the task is not fully completed. Because of this scoring system, the ARAT can identify some compensatory movements but does not differentiate them from other sources of movement abnormalities. In contrast, the FMMA-UE does not use this scoring system.

We anticipated that FMMA-UE and ARAT scores would initially align closely in the acute/early subacute phase, reflecting restoration-based recovery as the main driver behind both assessments. As recovery progresses beyond the subacute stage, we expected compensatory strategies to create a divergence between ARAT and FMMA-UE scores, with ARAT scoring allowing for further gains due to compensatory strategies for task completion (55). Thus, we expected decreased sensitivity and specificity of cut-off scores in the chronic phase after the restoration plateau. However, the consistent association between FMMA-UE and ARAT across different recovery stages suggests that these scores alone cannot adequately evaluate differences based on restoration versus compensation (55). Considering this, our findings reinforce recent recommendations advocating for the inclusion of kinematic assessments or explicit evaluations of motor control (5, 55). Such assessments could provide a more nuanced understanding of post-stroke recovery, thereby facilitating tailored rehabilitation strategies.

The primary limitation of this study was its reliance on data from previous studies, which, due to differing time points, were used for cross-sectionally analysis at each specified recovery stage. We compared the ability of FMMA-UE cut-off scores to predict ARAT-based functional categories at different stages of recovery. Additionally, while the established Minimal Clinically Important Difference (MCID) and Minimal Detectable Change (MDC) values provided useful benchmarks, they should be interpreted with caution. We utilized both metrics in our study to ensure a comprehensive analysis, acknowledging that these represent the best available measures under current methodological constraints.

In summary, our study revealed that FMMA-UE can classify upper extremity functional categories as defined by performance on the ARAT across all stages of stroke recovery. This is both scientifically relevant and clinically meaningful because it allows for a more standardized approach to evaluating post-stroke recovery. Additionally, the high congruence of both measures throughout different stages of recovery indicates that they are not suited to comprehensively capture the difference between restitution of impairment versus improvement. These findings emphasize the need to include assessments, such as kinematics, that allow for better measurement of recovery to enhance our understanding of upper limb functionality throughout rehabilitation.

## Data availability statement

The data analyzed in this study is subject to the following licenses/restrictions: the data analyzed in this study was obtained from the University of Zurich and cereneo Center for neurology and rehabilitation, the following licenses/restrictions apply: the dataset analyzed in this study is available upon reasonable request. Requests to access these datasets should be directed to AL, Department of Neurology, University Hospital Zurich, University of Zurich, Zurich, Switzerland, andreas.luft@usz.ch. Requests to access these datasets should be directed to AL, andreas.luft@usz.ch.

## Ethics statement

The studies involving humans were approved by the ethics committee of Zurich and the ethics committee of Northwest and Central Switzerland. The studies were conducted in accordance with the local legislation and institutional requirements. The participants provided their written informed consent to participate in this study.

## Author contributions

BV: Conceptualization, Data curation, Formal analysis, Investigation, Methodology, Project administration, Resources, Software, Validation, Visualization, Writing – original draft, Writing – review & editing. RK: Investigation, Methodology, Resources, Validation, Writing – review & editing. JP: Data curation, Investigation, Resources, Writing – review & editing. JH: Data curation, Investigation, Resources, Writing – review & editing. AL: Data curation, Funding acquisition, Investigation, Methodology, Project administration, Resources, Supervision, Writing – review & editing. JV: Conceptualization, Data curation, Investigation, Methodology, Resources, Writing – review & editing. MB: Conceptualization, Investigation, Methodology, Project administration, Resources, Supervision, Validation, Writing – original draft, Writing – review & editing.
