# Supplementary material for: The association between dexterity and upper limb impairment during stroke recovery
Source: Front Neurol. 2024 Aug 19;15:1429929. doi: 10.3389/fneur.2024.1429929 (PMC11367986; doi:10.3389/fneur.2024.1429929)
Supplement: Supplementary file 1 [file Table_1.DOCX]

Supplementary Material

# Supplementary Data

## Criteria for the three cohort studies:

In our study, we had one randomized controlled trial (Cohort 2) and two observational cohort clinical stroke recovery studies (Cohort 3 and 4). For Cohort 2, we analyzed data from all study visits during the subacute and chronic phases that had information. In Cohort 3, we focused on visits occurring at (10±2), (28±4), (90±7) and (365±14) days after stroke. Cohort 4 included data from visits at (7±1) and (90±10) days post-stroke.

**Cohort 2:**

Inclusion Criteria:

- Above 18 years of age with unilateral stroke and residual hemiparesis leading to a decrease of arm function after completion of all inpatient rehabilitation (at least 90 days post-stroke)
- Ability to lift arm against gravity (>30 degrees flexion or abduction)
- Ability to don/doff the devices on both wrists independently or with the assistance of a caregiver
- Ability to give informed consent as documented by signature

Exclusion Criteria:

- Major untreated depression
- Severe cognitive impairment
- Suffering from comprehensive aphasia
- Severely impaired sensation (unable to feel a soft touch on the dorsal side of their paretic wrist with closed eyes)
- Other major comorbidities (e.g., cardiopulmonary disease, renal failure, hepatic dysfunction, orthopaedic disorders, etc.)
- Expected hospitalization during the study period
- Known intolerance to device material
- Known or suspected non-compliance, drug or alcohol abuse
- The investigator, his/her family members, employees, and other dependent persons

Study design: In this cohort, patients underwent three study visits: visit 1 served as the baseline assessment, visit 2 took place after 6 weeks from the start of the study and served as the post-intervention evaluation, and visit 3 was a follow-up assessment conducted 12 weeks after the study commencement.

**Cohort 3:**

Inclusion Criteria:

- First-ever ischemic or hemorrhagic stroke, confirmed by MRI-DWI and/or CT (recurrent strokes are allowed when already included in this study after a first-ever stroke)
- Paresis or paralysis of the arm and/ or leg
- Living independently before stroke (mRS >2)
- Age 18 years or older
- Written informed consent of the patient or its legal representative after participants' information

Exclusion Criteria:

- Contra-indications on ethical grounds (vulnerable persons)
- Neurological or other diseases affecting upper limb use and/ or physical activity before stroke
- Known or suspected non-compliance, drug or alcohol abuse

Study design: This study included six study visits, scheduled on days 3±2, 10±2, 28±4, 90±7, and 365±14 poststroke, as well as at rehabilitation discharge.

**Cohort 4:**

Inclusion Criteria:

- First-ever unilateral ischemic stroke in the middle cerebral artery territory <48 hours, confirmed by MRI-DWI and/or CT
- Age 18 years or older
- Able to follow one-staged commands
- NIHSS arm score ≥1
- Informed consent after participants' information

Exclusion Criteria:

- Modified Rankin Scale score >2 before stroke
- Neurological or other diseases affecting the upper limb(s) before stroke
- Intravenous line in the upper limb(s), which limits assessment
- Contra-indications on ethical grounds
- Expected or known non-compliance to participate in the observational study, severe drug or/and alcohol abuse

Study design: Patients from Cohort 4 had three study visits. The first took place within 48 hours after symptom onset, the second on day 7±2 and the third on day 90±10 poststroke.

## Outcome measures

**Fugl-Meyer Motor Assessment for Upper Extremity**

The FMMA-UE is a test that was developed for patients of all ages who have experienced hemiplegia following a stroke.(11,53)

To perform the test, an armless chair with no wheels, a reflex hammer, a pencil, an index card, a tomato soup can, a tennis ball, and a stopwatch are required.

During the assessment, the participant should be seated in an upright position in the armless chair, and the test typically takes around 30 minutes to complete.

It is permissible to use verbal and mime instructions during the test, and the clinician should cue the participant immediately to limit the number of necessary repetitions.

It is assumed that the unaffected side has full passive and active range of motion and is without any impairment, while no considerations are made for the contracture of the affected side.

When performing the test, begin by having the patient perform the movement with their non-affected extremity first, and on the affected side, do not check for available passive range of motion.

Each movement should be requested to be performed up to three times on the affected side, and the best performance should be scored. However, if the full score is attained on trials 1 or 2, there is no need to repeat three times.

Coordination and speed are only tested once, and as a general rule, the patient should not be assisted unless noted otherwise. Verbal encouragement is permitted.

During the wrist tests, support can be provided under the elbow to decrease demand at the shoulder, but the patient should still activate the elbow flexors during the elbow at 90º tests, the elbow extensors during the elbow at 90º tests, and the elbow extensors during the elbow at 0º tests.

During the hand tests, assistance may be provided to position the arm by supporting the arm at the elbow and proximal to the wrist.

**Action Research Arm Test**

The assessment can be performed in two ways; testing only the items corresponding to the patient´s ability per Lyle´s instructions or testing all items.(12,35) Items are rated based on time and movement quality. Points are deducted for decreasing the quality of movement or taking more than the allotted time to complete the task. The equipment required is an armless chair with no wheels, a testing table, and an ARAT kit. The standardized ARAT kit consists of woodblocks of 2, 5, 7.5, and 10 cm3; a cricket ball; a sharpening stone; two alloy tubes, a washer, and a bolt; two plastic cups; a marble; a ball bearing; and a stopwatch. The complete assessment usually requires 20 minutes. Verbal encouragement is permitted. The assessor may give up to 3 attempts. A score of 3 is assigned when the task is completed in a standard manner (less than 5 seconds, maintaining proper body positioning, utilizing normal hand movements, and exhibiting normal arm movements). A score of 2 is assigned when the task is accomplished but with significant difficulty or an abnormal duration (abnormal hand, arm, or body posture). A score of 1 is awarded when the subject only partially completes the task. For grasp, grip, and pinch, the subject must have some hand movement to achieve 1. A score of 0 is given when the subject cannot complete any part of the hand or arm movement components within 60 seconds.

# Supplementary Figures and Tables

## Supplementary Figures

Supplementary Figure 1

**
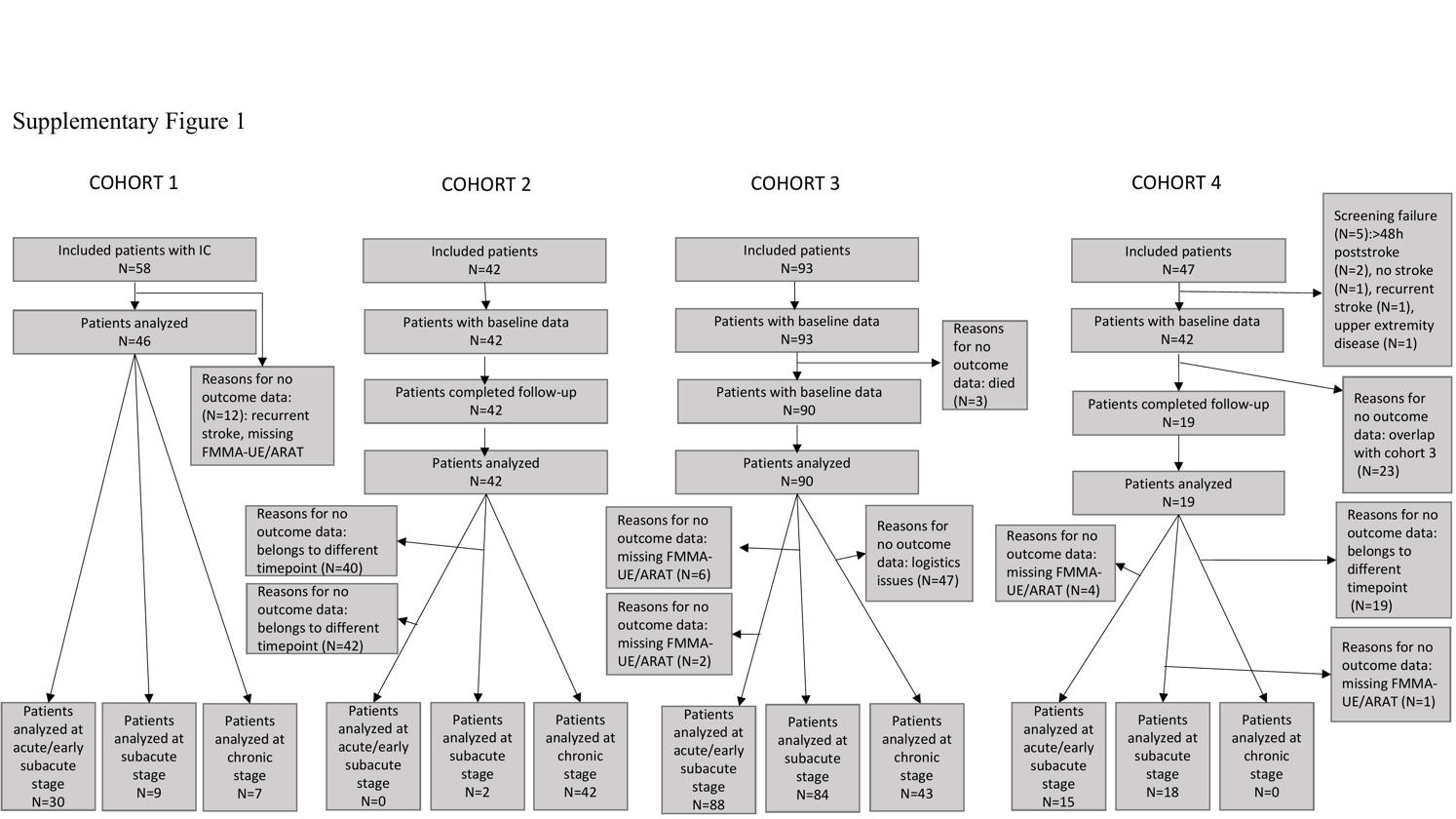
**

Supplementary Figure 1 displays the patient flow in the four cohorts for the primary research question. NOTE: In Cohort 2, no patients were in the acute/early subacute stage (2 weeks ±14), resulting in an absence of data for this timeframe (n=0). For the subacute stage (3-6 months±14), only 2 patients were enrolled, with the data for the remaining 40 patients being attributed to time points outside this specific stage, predominantly in the chronic stage (>6 months post-stroke). In Cohort 4, no patients were in the chronic stage (>6 months post-stroke), resulting in an absence of data for this timeframe (n=0). Abbreviations: IC, Informed consent.

## Supplementary Tables

Supplementary Table 1. STROBE Statement: Checklist of items that should be included in reports of cohort studies.

|  | | Item No | Recommendation | Page No |
| --- | --- | --- | --- | --- |
| **Title and abstract** | | 1 | (*a*) Indicate the study’s design with a commonly used term in the title or the abstract | 1, 2 |
|  |  |  | (*b*) Provide in the abstract an informative and balanced summary of what was done and what was found | 2 |
| Introduction | | | | |
| Background/rationale | | 2 | Explain the scientific background and rationale for the investigation being reported | 3-4 |
| Objectives | | 3 | State specific objectives, including any prespecified hypotheses | 4 |
| Methods | | | | |
| Study design | | 4 | Present key elements of study design early in the paper | 5 |
| Setting | | 5 | Describe the setting, locations, and relevant dates, including periods of recruitment, exposure, follow-up, and data collection | 5, 6 |
| Participants | | 6 | (*a*) Give the eligibility criteria, and the sources and methods of selection of participants. Describe methods of follow-up | 5,6 |
|  |  |  | (*b*) For matched studies, give matching criteria and number of exposed and unexposed |  |
| Variables | | 7 | Clearly define all outcomes, exposures, predictors, potential confounders, and effect modifiers. Give diagnostic criteria, if applicable | 5, 6 |
| Data sources/ measurement | | 8* | For each variable of interest, give sources of data and details of methods of assessment (measurement). Describe comparability of assessment methods if there is more than one group | 5,6 |
| Bias | | 9 | Describe any efforts to address potential sources of bias |  |
| Study size | | 10 | Explain how the study size was arrived at | 5,6 |
| Quantitative variables | | 11 | Explain how quantitative variables were handled in the analyses. If applicable, describe which groupings were chosen and why | 6 |
| Statistical methods | | 12 | (*a*) Describe all statistical methods, including those used to control for confounding | 6,7 |
|  |  |  | (*b*) Describe any methods used to examine subgroups and interactions |  |
|  |  |  | (*c*) Explain how missing data were addressed |  |
|  |  |  | (*d*) If applicable, explain how loss to follow-up was addressed |  |
|  |  |  | (*e*) Describe any sensitivity analyses | 6 |
| Results | | | |  |
| Participants | | 13* | (a) Report numbers of individuals at each stage of study—eg numbers potentially eligible, examined for eligibility, confirmed eligible, included in the study, completing follow-up, and analysed | 8, Table 1, 17, Supplementary Material Fig 1 |
|  |  |  | (b) Give reasons for non-participation at each stage | Supplementary Material Figure 1 |
|  |  |  | (c) Consider use of a flow diagram | Supplementary Material Figure 1 |
| Descriptive data | | 14* | (a) Give characteristics of study participants (eg demographic, clinical, social) and information on exposures and potential confounders | 8, Table 1, 17, |
|  |  |  | (b) Indicate number of participants with missing data for each variable of interest | Supplementary Material Figure 1 |
|  |  |  | (c) Summarise follow-up time (eg, average and total amount) |  |
| Outcome data | 15* | Report numbers of outcome events or summary measures over time | | 8, 9, Table 2, Table 3, table 4 |
| Main results | 16 | (*a*) Give unadjusted estimates and, if applicable, confounder-adjusted estimates and their precision (eg, 95% confidence interval). Make clear which confounders were adjusted for and why they were included | | 8,9 |
|  |  | (*b*) Report category boundaries when continuous variables were categorized | |  |
|  |  | (*c*) If relevant, consider translating estimates of relative risk into absolute risk for a meaningful time period | |  |
| Other analyses | 17 | Report other analyses done—eg analyses of subgroups and interactions, and sensitivity analyses | | 8,9, Table 4 |
| Discussion | | | | |
| Key results | 18 | Summarise key results with reference to study objectives | | 10 |
| Limitations | 19 | Discuss limitations of the study, taking into account sources of potential bias or imprecision. Discuss both direction and magnitude of any potential bias | | 11 |
| Interpretation | 20 | Give a cautious overall interpretation of results considering objectives, limitations, multiplicity of analyses, results from similar studies, and other relevant evidence | | 10,11 |
| Generalisability | 21 | Discuss the generalisability (external validity) of the study results | | 11 |
| Other information | | | | |
| Funding | 22 | Give the source of funding and the role of the funders for the present study and, if applicable, for the original study on which the present article is based | | Online submission system |

*Give information separately for exposed and unexposed groups.

**Note:** An Explanation and Elaboration article discusses each checklist item and gives methodological background and published examples of transparent reporting. The STROBE checklist is best used in conjunction with this article (freely available on the Web sites of PLoS Medicine at http://www.plosmedicine.org/, Annals of Internal Medicine at http://www.annals.org/, and Epidemiology at http://www.epidem.com/). Information on the STROBE Initiative is available at http://www.strobe-statement.org.
